# Implementation and training with laparoscopic distal pancreatectomy: 23-year experience from a high-volume center

**DOI:** 10.1007/s00464-021-08306-3

**Published:** 2021-02-03

**Authors:** Mushegh A. Sahakyan, Bård I. Røsok, Tore Tholfsen, Dyre Kleive, Anne Waage, Dejan Ignjatovic, Trond Buanes, Knut Jørgen Labori, Bjørn Edwin

**Affiliations:** 1 The Intervention Centre, Rikshospitalet, Oslo University Hospital, Oslo, Norway; 2 Department of Research & Development, Division of Emergencies and Critical Care, Oslo University Hospital, Oslo, Norway; 3 Department of Surgery N1, Yerevan State Medical University After M. Heratsi, Yerevan, Armenia; 4 Department of Hepato-Pancreato-Biliary Sugery, Rikshospitalet, Oslo University Hospital, Oslo, Norway; 5 Department of Digestive Surgery, Akershus University Hospital, Lørenskog, Norway; 6 Institute of Clinical Medicine, Faculty of Medicine, University of Oslo, Oslo, Norway

**Keywords:** Laparoscopy, Pancreatectomy, Conversion, Morbidity, Adenocarcinoma

## Abstract

**Background:**

Distal pancreatectomy is the most common procedure in minimally-invasive pancreatic surgery. Data in the literature suggest that the learning curve flattens after performing up to 30 procedures. However, the exact number remains unclear.

**Methods:**

The implementation and training with laparoscopic distal pancreatectomy (LDP) in a high-volume center were studied between 1997 and 2020. Perioperative outcomes and factors related to conversion were assessed. The individual experiences of four different surgeons (pioneer and adopters) performing LDP on a regular basis were examined.

**Results:**

Six hundred forty LDPs were done accounting for 95% of all distal pancreatectomies performed throughout the study period. Conversion was needed in 14 (2.2%) patients due to intraoperative bleeding or tumor adherence to the major vasculature. Overall morbidity and mortality rates were 35 and 0.6%, respectively. Intra- and postoperative outcomes did not change for any of the surgeons within their first 40 cases. Operative time significantly decreased after the first 80 cases for the pioneer surgeon and did not change afterwards although the proportion of ductal adenocarcinoma increased. Tumor size increased after the first 80 cases for the first adopter without affecting the operative time.

**Conclusions:**

In this nearly unselected cohort, no significant changes in surgical outcomes were observed throughout the first 40 LDPs for different surgeons. The exact number of procedures required to overcome the learning curve is difficult to determine as it seems to depend on patient selection policy and specifics of surgical training at the corresponding center.

**Supplementary Information:**

The online version of this article (10.1007/s00464-021-08306-3) contains supplementary material, which is available to authorized users.

The proportion of minimally-invasive pancreatic resections performed worldwide has been steadily increasing [1, 2]. Distal pancreatectomy is the most common procedure among those [3, 4]. Randomized controlled trials have shown clear advantages for minimally-invasive distal pancreatectomy (MIDP) over its open counterpart [5, 6]. As a result, current international guidelines recommend considering MIDP as a primary approach in patients with benign lesions and low-grade malignancies in the body and tail of the pancreas [2, 7, 8].

According to the literature, up to 30 procedures should be performed to overcome the learning curve and reach proficiency in MIDP [9–15]. However, interpretation of the literature data is difficult due to several reasons. First, findings from different studies are hardly comparable as they are based on both single- and multicenter experiences, as well as on single-surgeon series. Second, these reports often include a relatively small number of highly selected patients. Finally, analysis of pooled data from multiple surgeons with different levels of expertise in MIDP complicates the understanding of its true learning curve.

The aim of our report was to analyze 23-year experience with laparoscopic distal pancreatectomy (LDP) in a high-volume center for pancreatic surgery. The focus was on the implementation and training with this procedure for different surgeons.

## Materials and methods

### Study design

Study included a nearly unselected cohort of patients who underwent LDP at Oslo University Hospital, Rikshospitalet from April 1997 to June 2020. All patients were evaluated at the multidisciplinary team meeting prior to surgery and the final choices of management were made. The international guidelines were followed when deciding on the inclusion/exclusion criteria for surgery. Pancreatic cancer itself has never been an exclusion criteria. No preoperative histology was performed in these patients, i.e. those with radiological suspicion of cancer were considered for surgery. Since the pioneer surgeon performed the first LDP at our institution in April 1997, it has become a standard procedure for patients with lesions in the body and tail of the pancreas. Open distal pancreatectomy was reserved primarily for the patients with vascular involvement, where major vascular resection and reconstruction were needed. As a result, it was applied in only 5% of patients throughout the study period (Supplementary Fig. 1).

Four different surgeons performed the evaluated procedures. The pioneer surgeon performed his initial procedures in 1997 at the time of the advent of laparoscopic pancreatic surgery without receiving specific training or supervision. The first adopter was trained by the pioneer surgeon in mid-2000s and became an expert over time. During the last four years two trainees were trained by the pioneer and expert to be able to perform LDP on a regular basis. The expert and trainees all had broad experience in performing laparoscopic cholecystectomy, appendectomy, colon resection or gastric bypass/gastroenterostomy when initiating their learning curve in LDP. All three trainees received a multimodal surgical education including training during their fellowship in open pancreatic surgery, acting as the camera holder first during LDP and then performing LDP. The above-mentioned  four surgeons have done the largest number of LDPs at our institution accounting for 87.5% of these procedures performed throughout the study period. The extent of LDP and its technique have not significantly changed over time and the principles introduced by the pioneer surgeon have been followed by the adopters.

Information on patient demographics, comorbidities, and clinical characteristics, history of previous upper abdominal surgery, intraoperative parameters and postoperative results was retrieved from a prospectively maintained database. The experience with LDP was assessed. Procedures that were eventually converted to open surgery were also studied to identify specific factors leading conversion. Potential changes in perioperative results of LDP associated with an individual experience of the operating surgeon were assessed for the above-mentioned surgeons. The study was approved by the hospital review board according to the guidelines provided by the regional ethics committee.

The first 40 procedures of each surgeon were selected to examine the initial experience with LDP. Perioperative outcomes in these 40 cases were examined in 4 groups consisting of 10 patients each. The results of the first 40 LDPs were compared with the subsequent experience with this procedure (40 by 40). For better understanding of the individual results of each surgeon, the outcomes of the first 30 cases operated by the adopters without senior surgeon supervision/assistance were compared to those of a pioneer surgeon.

### Definitions

Previous upper abdominal surgery was defined as a surgical procedure performed in the upper portion of the peritoneal cavity, i.e. involving any organ located higher than the umbilicus. These included procedures on the hepato-pancreato-biliary system, stomach, spleen, small intestine, kidney, adrenal glands, upper retroperitoneum, and diaphragm. Colectomy involving the upper abdomen in the dissection area was considered as previous upper abdominal surgery.

Multivisceral resections included extended distal pancreatectomy and non-contiguous organ resection in the setting of distal pancreatectomy, as defined by the International Study Group for Pancreatic Surgery (ISGPS) [16]. Conversion was defined as laparotomy during LDP not related to the specimen extraction. Oslo classification based on Satava approach to surgical error evaluation was used to define and grade intraoperative adverse incidents [17, 18]. Postoperative morbidity was defined based on the Accordion Severity Grading System [19]. Grade ≥ III complications were considered severe. Postoperative pancreatic fistula (POPF) was reported according to the 2016 update from the ISGPS [20]. Postpancreatectomy hemorrhage was defined and classified as suggested by the ISGPS [21]. The 90 days from surgery definition was used for mortality and readmission [22].

### Technique

Patients are placed on a modified supine position with the left side raised 30°–45° [23, 24]. The first 12-mm trocar is placed through the umbilicus. Then 5-mm trocar is placed in the midline between the xiphoid process and the umbilicus. The third is 12-mm trocar (camera)—lateral to the left rectus muscle at the level of the umbilicus. Finally, one 5-mm trocar is placed in the left subcostal region, on the midclavicular line. For the centrally located pancreatic tumors the second trocar could be placed laterally from the right rectus muscle and above the umbilicus.

The procedure usually starts with the mobilization of splenic flexure of the colon followed by division of the short gastric vessels. However, the short gastric vessels are preserved if spleen-preserving LDP is performed. After releasing the transverse colon medially, the inferior margin of the pancreas is dissected free from the retroperitoneum. After the mobilization of the pancreatic body and tail is completed, and the splenic vessels are identified. In case of PDAC, the dissection plane is normally extended to the origin of the splenic artery and to the confluence of the splenic and superior mesenteric veins. Laparoscopic intraoperative ultrasound is often used to identify major vascular structures and examine their relationship with the lesion. The splenic vessels are divided by an Endo-GIA stapling device (Medtronic, Minneapolis, MA, USA). Regional lymph nodes (around the celiac trunk, along the splenic artery/inferior border of the pancreas and in the splenic hilum) are removed en bloc with the specimen. The level of pancreatic gland division depends on tumor location. Pancreatic body or neck is transected using a 60 mm EndoGIA stapling device (Medtronic, Minneapolis, MA, USA). After full mobilization of the spleen, the specimen is placed into the EndoCatch (Medtronic, Minneapolis, MA, USA) and retrieved through a small extension of the umbilical port incision. The use of the fibrinogen/thrombin-coated collagen sponge or glue on the pancreatic stump is left at surgeon’s discretion. One drain is routinely placed near the pancreatic stump.

### Statistics

The continuous data were expressed as mean (± standard deviation) or median (range) depending on data distribution. The analysis of variance (ANOVA) was used to compare normally distributed continuous data, and the post hoc test was used to verify statistically significant differences between the means. The Kruskal–Wallis and two-sided Mann–Whitney U-test were used for not normally distributed continuous data. The categorical variables were expressed as numbers (percentages). To compare these, the Chi-square test or Fisher’s exact test were. The two-sided *p*-value < 0.05 was considered statistically significant.

## Results

### Overview

A total number of 640 patients underwent LDP throughout the study period. Ductal adenocarcinoma was the indication for surgery in 138 (21.6%) patients (Table 1). The pioneer, expert, trainee 1 and trainee 2 performed 304 (47.5%), 169 (26.4%), 39 (6.1%) and 48 (7.5%) procedures, respectively. The remaining 80 (12.5%) patients were operated by other surgeons, mostly under the supervision of the pioneer or expert. Median operative time and blood loss were 160 min and 60 ml, respectively. Conversion to open surgery was needed in 14 (2.2%) patients. Postoperative complications developed in 224 (35%) patients including 119 (18.6%) with grade B/C pancreatic fistula. Reoperation was done in 34 (5.3%) cases, and median postoperative length of stay was 5 days.

**Table 1:** Experience with laparoscopic distal pancreatectomy at Oslo University Hospital, Rikshospitalet: 1997–2020

Parameters	*n* = 640
Age, years, mean (SD)	61 (14)
Body mass index, kg/m^2^, mean (SD)	25.8 (4.6)
Gender, *n* (%)	
Female/male	331 (51.7%)/309 (48.3%)
Cardiovascular diseases, *n* (%)	110 (17.4%)
Chronic obstructive pulmonary disease, *n* (%)	102 (15.9%)
Diabetes mellitus, *n* (%)	102 (15.9%)
Total number of comorbidities, mean (SD)	1.4 (1.3)
ASA score ≥ III, *n* (%)	203 (32%)
Diagnosis of PDAC, *n* (%)	138 (21.6%)
Tumor size, mm, median (range)	34 (4–180)
*Surgeons*, *n* (%)	
Pioneer	304 (47.5%)
Expert	169 (26.4%)
Trainee 1	39 (6.1%)
Trainee 2	48 (7.5%)
Other	80 (12.5%)
Spleen-preserving procedure, *n* (%)	91 (14.2%)
Multivisceral resection, *n* (%)	87 (13.6%)
Conversion, *n* (%)	14 (2.2%)
Operative time, min, median (range)	160 (30–560)
Estimated blood loss, ml, median (range)	60 (10–6250)
Perioperative red blood cell transfusion, *n* (%)	57 (9%)
*Postoperative complications*, *n* (%)	224 (35%)
Severe complications (grade ≥ III), *n* (%)	139 (21.7%)
*Clinically relevant pancreatic fistula*, *n* (%)	119 (18.6%)
Grade B/C hemorrhage, *n* (%)	33 (5.2%)
Reoperation, *n* (%)	34 (5.3%)
Readmission, *n* (%)	62 (9.7%)
90-day mortality, *n* (%)	4 (0.6%)
Postoperative length of stay, days, median (range)	5 (2–81)

*ASA* American Society of Anesthesiologists, *PDAC* pancreatic ductal adenocarcinoma

### Conversion

Detailed description of cases that required conversion to open surgery is given in Table 2. The majority of those (10 of 14) were operated for pancreatic cancer. In 9 cases, the operating surgeon had individual record of more than 40 LDPs performed. The reasons for conversion were adherence to the major vessels potentially requiring vascular resection and reconstruction (*n* = 8) and major intraoperative bleeding (*n* = 6). Major vascular resection with reconstruction was performed in 7 cases and multivisceral resection without vascular reconstruction was done in 3 patients. Eight patients developed postoperative complications.

**Table 2:** Description of cases that required conversion from laparoscopic to open distal pancreatectomy

No	Patient BMI (kg/m^2^)	Diagnosis	Tumor size (mm)	Surgeon’s case no	Reason of conversion	Open procedure	Postoperative Complications
1	25.2	Chronic pancreatitis	–	3	Intraoperative bleeding	DP	–
2	26.4	Serosal cystadenoma	60	43	Bleeding from the upper pole of the spleen	DP; left adrenalectomy; stomach resection	–
3	29.4	NEN	55	51	Bleeding from the splenic vein	DP; left adrenalectomy; stomach resection	Thrombosis of the SMV
4	25.8	PDAC	17	43	Tumor adherence to the SMV	DP; resection of the SMV	–
5	25.9	PDAC	88	44	Tumor adherence to the portal vein	DP; resection of the portal vein; liver resection	–
6	25.4	Adenosquamous carcinoma	85	54	Bleeding from the portal vein	DP; resection of the portal vein	PF; hemorrhage
7	24.4	PDAC	35	57	Tumor infiltration into the duodenum and adherence to the middle colic artery	DP; resection of the duodenum	PF
8	14.9	PDAC	55	59	Bleeding from the splenic vein	DP	–
9	35.2	PDAC	44	110	Bleeding from the coeliac trunk	DP; repair of the vascular injury	Pulmonary embolism; PF
10	26.2	PDAC	77	129	Tumor adherence to the portovenous confluence	DP; resection of the portal vein	Urinary incontinence
11	26.1	Adenosquamous carcinoma	70	24	Tumor adherence to the portal vein	DP; resection of the portal vein	–
12	38.9	Serosal cystadenoma	45	12	Tumor adherence to the SMV	DP; resection of the SMV	Hemorrhage; thrombosis of the SMV; PF
13	28.1	PDAC	9	19	Fibrosis in the surgical area, suspicion of vascular affection	DP	PF
14	33.2	PDAC	44	36	Suspicion of vascular affection	DP	Pulmonary embolism

*DP* distal pancreato-splenctomy, *NEN* neudoendocrine neoplasia, *SMV* superior mesenteric vein, *PDAC* pancreatic ductal adenocarcinoma, *PF* pancreatic fistula

### Implementation and training

The individual experience with the first 40 LDPs was analyzed for the pioneer surgeon (Table 3a). The body mass index of the first 10 patients was significantly higher compared to the rest (30.6 vs 24.6 vs 23.6 vs 24.6 kg/m^2^, *p* = 0.03), and the tumor size was significantly larger in the last 10 patients (23.5 vs 25 vs 19.5 vs 44.5 mm, *p* = 0.03). No statistically significant changes were detected in other perioperative parameters including operative time, blood loss, conversion, and complications. The results of the 40 cases operated by the pioneer surgeon were compared with the next 6 consecutive periods including 40 cases each (Table 3b). A significant decrease in median operative time was detected after the first 80 cases. Thereafter, it did not change. The proportion of PDAC significantly increased, and the number of spleen-preserving LDP decreased in the later periods. Other intra- and postoperative parameters remained similar.

**Table 3:** Experiences of the pioneer and expert surgeons with LDP: first 40 cases of the pioneer (a); first and subsequent 40 cases of the pioneer (b); first 40 cases of the expert (c); first and subsequent 40 cases of the expert (d)

Parameters	1–10	11–20	21–30	31–40	*p*-value
(a)					
Age, years, mean (SD)	56.7 (12.2)	56 (15.2)	62.3 (10)	59.8 (12.9)	0.41
BMI, kg/m^2^, mean (SD)^a^	30.6 (7.7)	24.6 (1.9)	23.6 (3.2)	24.6 (3.3)	**0.03**
PUAS, *n*	4	6	6	3	0.54
Diagnosis (PDAC), *n*	1	2	2	2	1.0
Tumor size, mm, median (range)^b^	23.5 (4–70)	25 (11–75)	19.5 (15–23)	44.5 (29–70)	**0.03**
Spleen-preserving procedure, *n*	1	2	4	1	0.46
Multivisceral resection, *n*	0	2	2	1	0.73
Blood loss, ml, median (range)	100 (30–1000)	300 (30–1500)	80 (30–350)	250 (30–3000)	0.5
Operative time, min, median (range)	248 (125–360)	233 (180–520)	215 (123–300)	188 (135–260)	0.16
Intraoperative adverse events, *n*	2	3	0	2	0.46
Conversion, *n*	1	0	0	0	1.0
Postoperative complications, *n*	3	6	4	4	0.68
Severe complications, *n*	3	3	2	2	1.0
CR-PF, *n*	2	2	0	1	0.73
Grade B/C hemorrhage, *n*	1	1	0	1	1.0
Reoperation, *n*	0	0	0	1	1.0
Mortality, *n*	0	0	0	0	–
Hospital stay, days, median (range)	6 (4–8)	6 (2–12)	6 (2–11)	5 (3–16)	0.73

Parameters	Period 1 (1–40)	Period 2 (41–80)	Period 3 (81–120)	Period 4 (121–160)	Period 5 (161–200)	Period 6 (201–240)	Period 7 (241–280)	*p*–value
(b)								
Age, years, mean (SD)	60 (13)	57 (17)	55 (18)	55 (17)	60 (14)	64 (11)	66 (11)	**0.003**
BMI, kg/m^2^, mean (SD)	25.5 (4.9)	25.7 (4.9)	24.9 (4.2)	25.3 (4.9)	26.3 (5.2)	26.7 (5.6)	26.3 (4.7)	0.66
PUAS, *n* (%)	19 (47.5%)	16 (40%)	15 (37.5%)	20 (50%)	12 (30%)	20 (50%)	15 (37.5%)	0.46
ASA ≥ III, *n* (%)	14 (35%)	9 (22.5%)	8 (20%)	12 (30%)	15 (37.5%)	17 (42.5%)	10 (25%)	0.25
Diagnosis (PDAC), *n* (%)	7 (17.5%)	3 (7.5%)	4 (10%)	8 (20%)	15 (37.5%)	10 (25%)	15 (37.5%)	**0.003**
Tumor size, mm, median (range)	27 (4–75)	40 (15–110)	33 (8–110)	38 (10–115)	39 (12–130)	29 (8–85)	35 (11–145)	0.07
Spleen-preserving procedure, *n* (%)	8 (20%)	15 (37.5%)	5 (12.5%)	13 (32.5%)	1 (2.5%)	1 (2.5%)	2 (5%)	**0.001**
Subtotal distal pancreatectomy, *n* (%)	2 (5%)	12 (30%)	5 (12.5%)	7 (17.5%)	11 (27.5%)	6 (15%)	9 (22.5%)	0.06
Multivisceral resection, *n* (%)	5 (12.5%)	6 (15%)	4 (10%)	10 (25%)	8 (20%)	3 (7.5%)	10 (25%)	0.19
Blood loss, ml, median (range)	180 (30–3000)	230 (10–6250)	50 (30–2700)	100 (30–2100)	50 (30–1600)	200 (20–2000)	100 (30–3000)	0.23
Operative time, min, median (range)	220 (123–520)	198 (107–480)	158 (30–268)	132 (56–319)	147 (30–386)	143 (45–339)	142 (62–303)	** < 0.001**
Intraoperative adverse events, *n* (%)	7 (17.5%)	7 (17.5%)	2 (5%)	3 (7.5%)	2 (5%)	9 (22.5%)	4 (10%)	0.1
Conversion, *n* (%)	1 (2.5%)	2 (5%)	0 (0%)	0 (0%)	0 (0%)	0 (0%)	0 (0%)	0.38
Postoperative complications, *n* (%)	17 (42.5%)	9 (22.5%)	10 (25%)	17 (42.5%)	16 (40%)	14 (35%)	13 (32.5%)	0.32
Severe complications, *n* (%)	10 (25%)	6 (15%)	6 (15%)	11 (27.5%)	9 (22.5%)	6 (15%)	7 (17.5%)	0.64
CR-PF, *n* (%)	5 (12.5%)	6 (15%)	2 (5%)	7 (17.5%)	11 (27.5%)	9 (22.5%)	4 (10%)	0.11
Grade B/C hemorrhage, *n* (%)	3 (7.5%)	0 (0%)	2 (5%)	3 (7.5%)	3 (7.5%)	4 (10%)	2 (5%)	0.57
Reoperation, *n* (%)	1 (2.5%)	0 (0%)	2 (5%)	3 (7.5%)	5 (12.5%)	1 (2.5%)	4 (10%)	0.19
Mortality, *n* (%)	0 (0%)	0 (0%)	1 (2.5%)	0 (0%)	1 (2.5%)	1 (2.5%)	0 (0%)	1.0
Hospital stay, days, median (range)	6 (2–16)	6 (2–30)	5 (3–32)	6 (2–81)	5 (3–34)	5 (2–22)	5 (3–18)	0.38

Parameters	1–10	11–20	21–30	31–40	*p*-value
(c)					
Age, years, mean (SD)	58 (12.4)	65.7 (6.3)	60 (13)	57.6 (13.2)	0.39
BMI, kg/m^2^, mean (SD)	25.2 (5.5)	24.1 (3.1)	25.8 (3.5)	25.9 (4.3)	0.76
PUAS, *n*	3	4	3	5	0.89
Diagnosis (PDAC), *n*	1	0	1	3	0.36
Tumor size, mm, median (range)	30 (8–45)	28 (15–55)	28 (10–70)	23 (7–50)	0.53
Pioneer surgeon assistance/supervision, *n*	10	3	2	1	** < 0.01**
Spleen-preserving procedure, *n*	4	2	3	6	0.38
Multivisceral resection, *n*	1	0	2	1	0.89
Blood loss, ml, median (range)	70 (30–800)	50 (30–400)	75 (30–450)	100 (30–1200)	0.19
Operative time, min, median (range)	140 (115–205)	193 (80–356)	176 (110–292)	187 (110–284)	0.32
Intraoperative adverse events, *n*	1	0	0	2	0.6
Conversion, *n*	0	0	0	0	–
Postoperative complications, *n*	2	6	6	4	0.22
Severe complications, *n*	2	4	3	3	0.96
CR-PF, *n*	2	3	2	3	1.0
Grade B/C hemorrhage, *n*	0	2	2	0	0.3
Reoperation, *n*	0	1	2	0	0.6
Mortality, *n*	0	0	0	0	–
Hospital stay, days, median (range)	5 (2–13)	8 (5–35)	7 (2–9)	5 (3–11)	0.11

Parameters	Period 1 (1–40)	Period 2 (41–80)	Period 3 (81–120)	Period 4 (121–160)	*p*-value
(d)					
Age, years, mean (SD)	60 (12)	63 (12)	64 (12)	62 (14)	0.63
BMI, kg/m^2^, mean (SD)	25.2 (4.1)	24.5 (3.9)	25.8 (4.6)	26.5 (4.6)	0.19
PUAS, *n* (%)	15 (37.5%)	19 (47.5%)	16 (40%)	18 (45%)	0.8
ASA ≥ III, *n* (%)	13 (32.5%)	10 (25%)	12 (30%)	12 (30%)	0.9
Diagnosis (PDAC), *n* (%)	5 (12.5%)	12 (30%)	10 (25%)	9 (22.5%)	0.29
Tumor size, mm, median (range)	28 (7–70)	22 (7–88)	42 (8–127)	40 (12–130)	**0.002**
Pioneer surgeon assistance/supervision, *n*	16 (40%)	1 (2.5%)	2 (5%)	0 (0%)	** < 0.01**
Spleen-preserving procedure, *n* (%)	15 (37.5%)	5 (12.5%)	5 (12.5%)	6 (15%)	**0.01**
Subtotal distal pancreatectomy, *n* (%)	5 (12.5%)	13 (32.5%)	10 (25%)	11 (27.5%)	0.19
Multivisceral resection, *n* (%)	4 (10%)	8 (20%)	6 (15%)	4 (10%)	0.51
Blood loss, ml, median (range)	160 (30–1200)	150 (30–2500)	180 (30–2800)	170 (30–1600)	0.27
Operative time, min, median (range)	175 (80–356)	162 (92–503)	165 (91–428)	166 (74–560)	0.78
Intraoperative adverse events, *n* (%)	3 (7.5%)	6 (15%)	4 (10%)	3 (7.5%)	0.76
Conversion, *n* (%)	0 (0%)	5 (12.5%)	1 (2.5%)	1 (2.5%)	0.07
Postoperative complications, *n* (%)	18 (45%)	13 (32.5%)	14 (35%)	15 (37.5%)	0.68
Severe complications, *n* (%)	12 (30%)	7 (17.5%)	10 (25%)	8 (20%)	0.56
CR-PF, *n* (%)	10 (25%)	8 (20%)	5 (12.5%)	7 (17.5%)	0.55
Grade B/C hemorrhage, *n* (%)	4 (10%)	2 (5%)	2 (5%)	2 (5%)	0.84
Reoperation, *n* (%)	3 (7.5%)	1 (2.5%)	4 (10%)	3 (7.5%)	0.69
Mortality, *n* (%)	0 (0%)	1 (2.5%)	0 (0%)	0 (0%)	1.0
Hospital stay, days, median (range)	6 (2–35)	5 (2–31)	5 (2–44)	5 (3–29)	0.4

*BMI* body mass index, *PUAS* previous upper abdominal surgery, *PDAC* pancreatic ductal adenocarcinoma, *CR-PF* clinically relevant pancreatic fistula

^a^Significant difference between the periods 1 and 2–4

^b^Significant difference between the periods 1–3 and 4

The expert surgeon performed the first LDP after having assisted the pioneer on 9 LDPs. The experience with first 40 cases was analyzed (Table 3c). No significant changes in perioperative outcomes were observed although the number of cases assisted by the pioneer surgeon significantly decreased (10 vs 3 vs 2 vs 1, *p* < 0.01). No conversions were performed in this period. The analysis of the next 3 consecutive periods including 40 procedures each demonstrated significant increase in tumor size following the first 80 procedures (Table 3d). The proportion of spleen-preserving procedures decreased after period 1. Operative time did not change over time. The number of cases assisted/supervised by the pioneer surgeon decreased from 40% in the period 1 to 2.5%, 5% and 0% in the periods 2, 3 and 4, respectively (*p* < 0.01). Intra- and postoperative outcomes were comparable.

Before performing their first LDP trainees 1 and 2 had assisted on 11 and 7 procedures, respectively. Shorter time span was required for the trainees to perform their first 30–40 LDP compared with the pioneer and the expert (Supplementary Fig. 2). Their initial experience with LDP is presented in Table 4a and b. The number of cases assisted/supervised by either the pioneer or expert surgeons significantly decreased over time. Operative time and other perioperative outcomes did not change.

**Table 4:** The initial experience of the trainees with laparoscopic distal pancreatectomy: trainee 1 (a) and trainee 2 (b)

Parameters	1–10	11–20	21–30	31–39	*p*-value
(a)					
Age, years, mean (SD)	62.8 (12.3)	61.7 (13.7)	59 (15.5)	57.4 (14.3)	0.83
BMI, kg/m^2^, mean (SD)	25.2 (3)	28.7 (6.1)	27.4 (5.3)	28.3 (6)	0.45
PUAS, *n*	3	5	4	5	0.77
Diagnosis (PDAC), *n*	0	1	1	2	0.43
Tumor size, mm, median (range)	47 (11–114)	47.5 (20–95)	35 (16–60)	50 (27–80)	0.37
Pioneer/expert surgeon assistance/supervision, *n*	5	6	3	0	**0.025**
Spleen-preserving procedure, *n*	0	1	0	0	1.0
Multivisceral resection, *n*	0	1	1	1	0.89
Blood loss, ml, median (range)	50 (30–300)	125 (50–3700)	50 (20–250)	100 (10–400)	0.27
Operative time, min, median (range)	149 (100–202)	162 (125–341)	159 (78–314)	188 (122–219)	0.36
Intraoperative adverse events, *n*	0	3	0	2	0.08
Conversion, *n*	0	1	0	0	1.0
Postoperative complications, *n*	5	3	3	1	0.4
Severe complications, *n*	4	2	2	1	0.56
CR-PF, *n*	2	2	2	1	1.0
Grade B/C hemorrhage, *n*	1	1	1	0	1.0
Reoperation, *n*	0	1	1	0	1.0
Mortality, *n*	0	0	0	0	–
Hospital stay, days, median (range)	7 (4–19)	4 (3–38)	6 (3–13)	4 (3–7)	0.08

Parameters	1–10	11–20	21–30	31–40	*p*-value
(b)					
Age, years, mean (SD)	68.3 (6.8)	60 (16.3)	66.4 (8.1)	67.2 (11.1)	0.36
BMI, kg/m^2^, mean (SD)	26.5 (4.8)	26.9 (2.9)	27.3 (3.3)	28 (4.3)	0.85
PUAS, *n*	4	5	5	7	0.68
Diagnosis (PDAC), *n*	1	3	4	3	0.6
Tumor size, mm, median (range)	22 (14–37)	25 (9–100)	35 (13–80)	30 (11–110)	0.38
Pioneer/expert surgeon assistance/supervision, *n*	7	4	5	0	**0.008**
Spleen-preserving procedure, *n*	1	0	1	0	1.0
Multivisceral resection, *n*	0	0	1	0	1.0
Blood loss, ml, median (range)	50 (30–500)	50 (30–800)	50 (30–200)	50 (20–500)	0.88
Operative time, min, median (range)	165 (95–267)	186 (148–325)	156 (71–389)	139 (76–341)	0.07
Intraoperative adverse events, *n*	0	2	0	1	0.6
Conversion, *n*	0	1	0	1	1.0
Postoperative complications, *n*	1	3	2	4	0.62
Severe complications, *n*	0	3	0	2	0.14
CR-PF, *n*	0	3	0	2	0.14
Grade B/C hemorrhage, *n*	0	0	0	0	–
Reoperation, *n*	0	0	0	1	1.0
Mortality, *n*	0	0	0	0	–
Hospital stay, days, median (range)	4 (2–5)	5 (3–40)	5 (3–6)	5 (3–9)	0.19

*BMI* body mass index, *PUAS* previous upper abdominal surgery, *PDAC* pancreatic ductal adenocarcinoma, *CR-PF* clinically relevant pancreatic fistula

The individual experiences of the adopters (expert and trainees) with their first 30 non-supervised LDPs were compared to the outcomes of the first 30 consecutive LDPs performed by the pioneer surgeon (Table 5). To reach a total number of 30 non-supervised LDPs, 46, 39 and 48 cases were required for the expert, trainee 1 and 2, respectively. These included procedures assisted or supervised by a senior surgeon.

**Table 5:** Experiences with the first 30 laparoscopic distal pancreatectomies for the pioneer and the adopters without senior surgeon assistance

Parameters	Pioneer	Expert	Trainee 1	Trainee 2	*p*-value
Within individual LDP series	1–30	1–46	1–39	1–48	
Age, years, mean (SD)	58.6 (13.7)	63.6 (10.7)	60.1 (13.3)	63.4 (12.1)	0.33
BMI, kg/m^2^, mean (SD)	25.9 (5.4)	24.8 (3.3)	27.4 (4.8)	26.5 (3.9)	0.13
ASA ≥ III, *n* (%)	10 (33.3%)	11 (36.7%)	10 (33.3%)	13 (43.3%)	0.72
PUAS, *n* (%)	16 (53.3%)	14 (46.7%)	14 (46.7%)	15 (50%)	0.95
Diagnosis (PDAC), *n* (%)	5 (16.7%)	7 (23.3%)	3 (10%)	8 (26.7%)	0.37
Tumor size, mm, median (range)	25 (4–80)	25 (7–88)	37 (13–80)	31 (11–110)	0.15
Spleen-preserving procedure, *n* (%)	7 (23.3%)	11 (36.7%)	1 (3.3%)	1 (3.3%)	**0.001**^┼^
Multivisceral resection, *n* (%)	4 (13.3%)	6 (20%)	1 (3.3%)	0 (0%)	**0.02**^╪^
Blood loss, ml, median (range)	120 (30–1500)	50 (30–900)	80 (10–400)	50 (20–750)	0.2
Operative time, min, median (range)	230 (123–520)	188 (110–356)	161 (78–314)	156 (71–341)	< 0.001*
Intraoperative adverse events, *n* (%)	5 (16.7%)	3 (10%)	3 (10%)	3 (10%)	0.89
Conversion, *n* (%)	1 (3.3%)	2 (6.7%)	0 (0%)	1 (3.3%)	0.9
Postoperative complications, *n* (%)	13 (43.3%)	11 (36.7%)	8 (26.7%)	9 (30%)	0.54
Severe complications, *n* (%)	8 (26.7%)	7 (23.3%)	7 (23.3%)	5 (16.7%)	0.87
CR-PF, *n* (%)	4 (13.3%)	5 (16.7%)	4 (13.3%)	4 (13.3%)	1.0
Grade B/C hemorrhage, *n* (%)	2 (6.7%)	3 (10%)	2 (6.7%)	1 (3.3%)	0.96
Reoperation, *n* (%)	0 (0%)	2 (6.7%)	1 (3.3%)	1 (3.3%)	0.9
Mortality, *n* (%)	0 (0%)	0 (0%)	0 (0%)	0 (0%)	–
Readmission, *n* (%)	3 (10%)	3 (10%)	4 (13.3%)	4 (13.3%)	1.0
Hospital stay, days, median (range)	6 (2–12)	6 (2–35)	5 (3–19)	4 (2–40)	**0.02**^**¶**^

*BMI* body mass index, *PUAS* previous upper abdominal surgery, *PDAC* pancreatic ductal adenocarcinoma, *CR-PF* clinically relevant pancreatic fistula

^┼^Significant difference between the pioneer/expert and trainees

^╪^Significant difference between the expert and trainees

*Significant difference between the pioneer and others

^¶^Significant difference between the pioneer/expert and trainee 2

Preoperative parameters such as patient demographics, body mass index, history of previous upper abdominal surgery, tumor size and diagnosis of PDAC was similar for all surgeons. The rate of spleen preserving procedures was significantly higher for the pioneer and expert compared to the trainees—23.3 vs 36.7 vs 3.3 vs 3.3%, (*p* = 0.001), respectively. The proportion of multivisceral resections was significantly higher in expert’s experience compared with the trainees—20 vs 3.3 vs 0% (*p* = 0.015), respectively. Median operative time significantly decreased when comparing the outcomes of the pioneer and adopters – 238 vs 188 vs 161 vs 156 min (*p* < 0.001). The incidences of intraoperative adverse events and conversion were similar. Postoperative outcomes including complications, pancreatic fistula, hemorrhage, reoperation and readmission were comparable. None of the patients died within 90 days of surgery. Median length of postoperative stay was significantly shorter for the trainee 2 compared with the pioneer and expert (4 vs 6 vs 6 (*p* = 0.02), respectively).

## Discussion

Our experience with LDP is based on its implementation and standardization of intraoperative steps by a pioneer surgeon followed by the stepwise training for the adopters. The consecutive phases of training included assisting the pioneer surgeon (and learning the intraoperative steps) (i), performing surgery with (ii) and without supervision (iii). As a result, satisfactory results were registered for the adopters. Interestingly, the conversion rate in this series was only 2%. In contrast, recent data from the national registries and high-volume pancreatic centers report 15–20% and 15–19% conversion for MIDP, respectively [25–29]. In half of our patients, conversion was necessary due to the need for major vascular resection and reconstruction, while in the rest, either intraoperative bleeding or suspicion of major vascular involvement was present. This suggests that, in our hands, vascular affection and major intraoperative bleeding were the main predictors of conversion in LDP.

Another important finding of this report was that no significant changes in intra- and postoperative outcomes was observed throughout the first 40 LDPs performed by each surgeon. Furthermore, 9 of 14 conversions reported occurred in surgeons who had already surpassed the experience with the first 40 cases. This is in contrast with the data in the literature suggesting improvement in operative time, estimated blood loss and conversion rate following 10–20 LDPs [9–11, 14, 26]. When analyzing the experience with LDP following the first 40 cases, significant reduction in operative time was demonstrated after 80 cases for the pioneer surgeon. The same trend was not present for the first adopter (expert) although tumor size significantly increased after the first 80 cases without significantly affecting the intra- and postoperative outcomes. The fact that the proportion for PDAC and tumor size increased for the pioneer and expert over time likely led to a decrease in utilization of spleen-preserving LDP. Analysis of the first 40 procedures performed by the adopters demonstrated steady decrease of senior surgeon assistance/supervision without compromising the intra- and postoperative outcomes. These findings suggest smooth transition for the adopters as their results did not significantly change when the oversight from senior surgeons was stopped, and more complex cases were undertaken.

Single-surgeon series published by de Rooij and co-workers suggest improvement in Clavien-Dindo grade ≥ III complications, grade B/C pancreatic fistula and hospital stay following the first 30 cases [12]. These findings were confirmed also in the multicenter study from 11 tertiary referral centers in the UK [15]. In that study, though, the minimally-invasive approach was applied in only half of the patients undergoing distal pancreatectomy. In contrast, there was almost no patient selection in our center as 95% of distal pancreatectomies were performed laparoscopically. No significant changes in postoperative outcomes were observed for any of the four above mentioned throughout the study period. This is in line with the report from Malleo et al., although they used LDP mainly for benign and low-grade malignancies [30].

Evaluation of the first 30 LDPs performed by the adopters without senior surgeon supervision demonstrated reduced operative time compared to the results of the pioneer surgeon. Superior outcomes for the trainees have also been demonstrated by Nakamura and co-workers [31]. However, one should consider that, in our study, in contrast with the pioneer the adopters had assisted 7–11 LDPs before undertaking their first procedure. Furthermore, slightly less than a half of their first 40 cases were assisted/supervised by a senior surgeon. Finally, less multivisceral and spleen-preserving procedures were performed by the trainees compared to the pioneer and expert indicating patient selection in the early phases of their learning curve.

This study has several strengths and limitations. One of the main advantages that distinguishes this study from similar publications is that it depicts the individual performance of 4 surgeons with different background and experience in a high-volume center. Furthermore, their outcomes with LDP was analyzed in different stages of their learning curves. Another advantage of this report is based on a large material encompassing more than 20 years of experience with this procedure. Most importantly, 95% of our patients were referred to LDP, which significantly reduces the risk of selection bias. The main drawback remains the retrospective observational design of this study with all inherent biases. Nearly 13% of the patients (*n* = 80) were not analyzed as they were operated by other surgeons at the department. At the same time, the majority (64%) of those procedures were assisted and supervised by the pioneer or expert. Furthermore, the postoperative outcomes of these 80 cases were not significantly different from those demonstrated by surgeons included in this study (data not shown).

Our findings suggest that standardization LDP technique and stepwise training in a high-volume center can significantly reduce the conversion rate providing satisfactory results for different surgeons. No significant improvement in perioperative outcomes of LDP has been detected throughout the first 40 LDPs of each surgeon. Furthermore, 80 procedures seem to be more realistic in the setting when almost no patient selection is present. The exact number of procedures required to overcome the learning curve is difficult to determine as it seems to depend on patient selection policy and surgical training program at the corresponding center.

## Supplementary Information

Supplementary figure 1. Experience with laparoscopic and open distal pancreatectomy throughout the study period. Supplementary Information 1 (JPG 39 kb)

Supplementary figure 2. The individual volumes of different surgeons with LDP and time period needed to achieve 40 procedures througout the study period. Supplementary Information 2 (JPG 36 kb)
