# Supplementary figures and images for: Implementation and training with laparoscopic distal pancreatectomy: 23-year experience from a high-volume center
Source: Surg Endosc. 2021 Feb 3;36(1):468–79. doi: 10.1007/s00464-021-08306-3 (PMC8741682; doi:10.1007/s00464-021-08306-3)

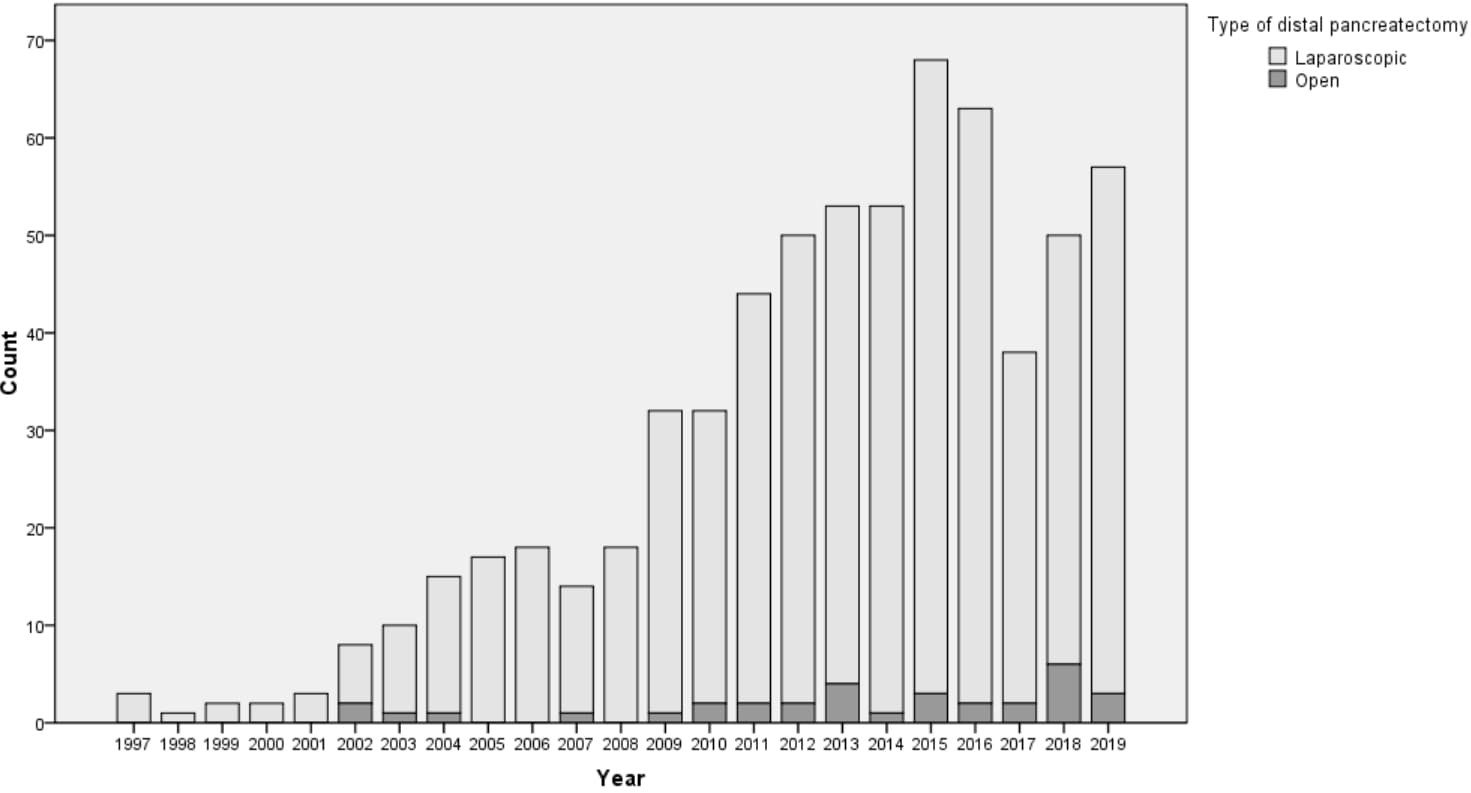

Supplement: Supplementary file 1 — Supplementary figure 1. Experience with laparoscopic and open distal pancreatectomy throughout the study period. Supplementary Information 1 (JPG 39 kb) [file 464_2021_8306_MOESM1_ESM.jpg]

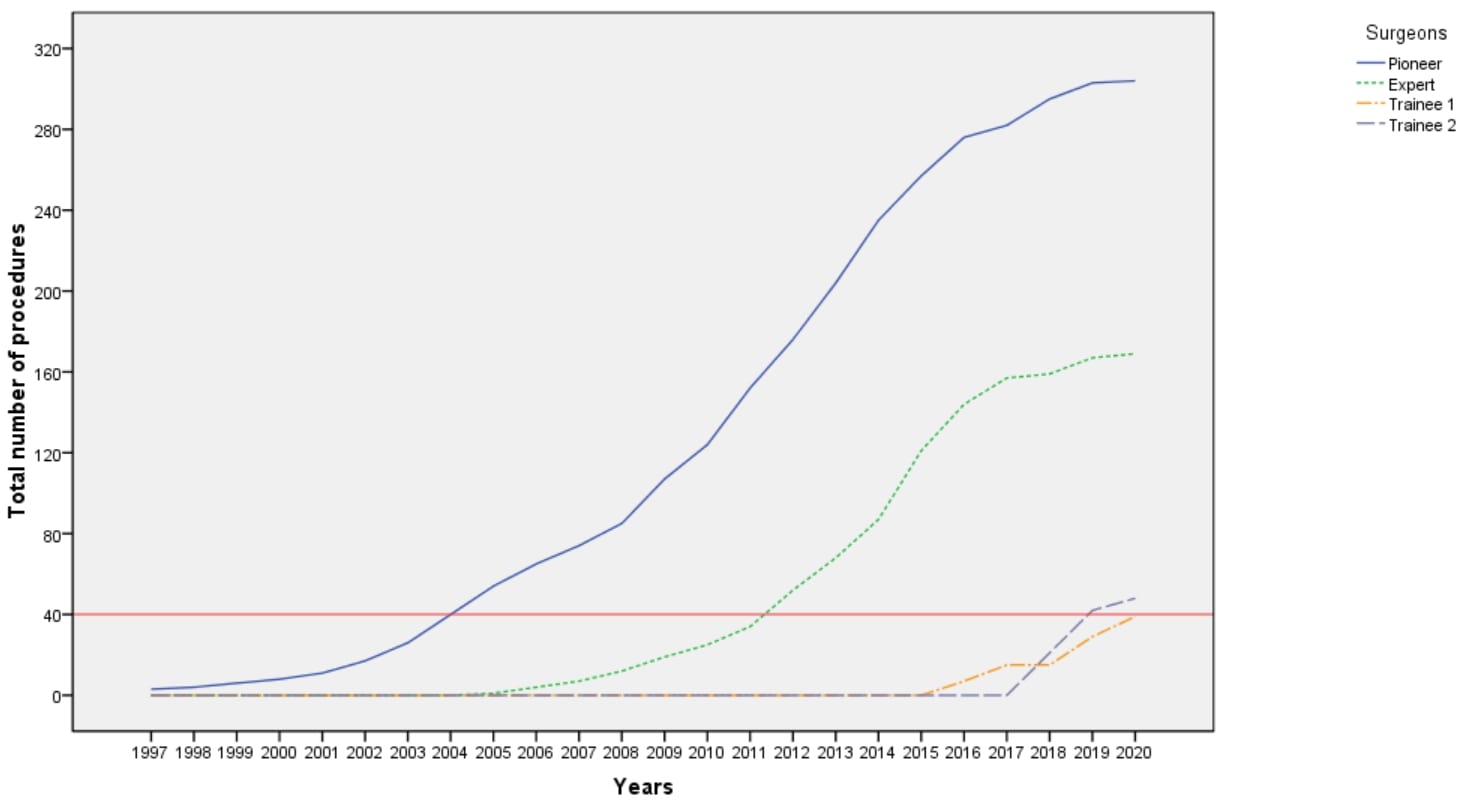

Supplement: Supplementary file 2 — Supplementary figure 2. The individual volumes of different surgeons with LDP and time period needed to achieve 40 procedures througout the study period. Supplementary Information 2 (JPG 36 kb) [file 464_2021_8306_MOESM2_ESM.jpg]
